# A randomized controlled study to evaluate the effect of pharmacist-led educational intervention on glycemic control, self-care activities and disease knowledge among type 2 diabetes patients: A consort compliant study protocol

**DOI:** 10.1097/MD.0000000000009847

**Published:** 2018-03-23

**Authors:** Allah Bukhsh, Muhammad Sarfraz Nawaz, Hafiz Sajjad Ahmed, Tahir Mehmood Khan

**Affiliations:** a School of Pharmacy, Monash University, Jalan Lagoon, Selatan, Selangor, Malaysia; b Institute of Pharmaceutical Sciences, University of Veterinary and Animal Sciences, Lahore; c Department of Pharmacy, Quaid-i-Azam University; d Capital Hospital Islamabad, Pakistan; e Asian Centre for Evidence Synthesis in Population, Implementation and Clinical Outcomes (PICO), Health and Well-being Cluster, Monash University Malaysia, Bandar Sunway, Selangor, Malaysia.

**Keywords:** HbA1c, pakistan, self-care, self-management, type 2 diabetes

## Abstract

**Background:**

Diabetes self-care activities, like, healthy diet, regular exercise, self-monitoring of blood glucose, and rational use of medicines are considered to play a vital role in establishing euglycemia. Health literacy among type 2 diabetes mellitus (T2DM) patients in Pakistan is very low, which is the most likely cause for poor clinical outcomes. This study is designed to investigate the impact of pharmacist-led educational intervention on glycemic control, self-care activities and disease knowledge among T2DM patients in Pakistan.

**Methods:**

In this randomized controlled trail, effectiveness of a 6-month pharmacist-led educational intervention will be examined on glycemic control, diabetes self-care activities and disease knowledge of 80 adult T2DM patients (age >30 years) with poorly controlled T2DM (HbA1c> 7%), after randomizing them into intervention and control groups, at diabetes care clinic of Capital Hospital Islamabad, Pakistan.

**Results:**

The primary outcome is change in patients’ HbA1c, whereas, changes in self-care activities and patients’ disease knowledge are the secondary outcomes. After baseline assessment of their self-care activities and disease knowledge by using validated Urdu versions of Diabetes Self-management Questionnaire (DSMQ) and Diabetes Knowledge Questionnaire (DKQ), respectively, interventional group patients will be supplemented with a face-to-face pharmacist-led educational intervention, whereas, the control group will receive usual care. Intervention arm patients will be educated successively at their first follow-up visit (12th week) and telephonically after every 4 weeks. All assessments will be made at baseline and end of trail for both intervention and control groups. Multivariate general linear model will be applied to analyze the effects of the intervention.

**Conclusion:**

Glycemic control in T2DM patients requires optimum self-care activities. This study is an attempt to improve self-care behaviors among poorly controlled T2DM patients who are at higher risk of diabetes-associated late complications.

## 1 Introduction

Globally, diabetes has become a serious clinical and public health problem. According to World Health Organization (WHO), the diabetes number increased from 108 million in 1980 to 422 million in 2014.^[1]^ Diabetes is the leading cause of kidney failure, stroke, heart attack, blindness, and lower limb amputations, and when not properly managed, it will lead to higher health care cost, morbidity, and mortality, thereby creating greater financial burdens.^[2,3]^ With rapidly rising prevalence of diabetes, especially in the developing countries, it is projected to be the 7th leading cause of mortality in 2030.^[1,3]^ Worldwide approximately 415 million people have been diagnosed with diabetes mellitus (DM), and the number is expected to increase to 642 million by 2040,^[4]^ making it one of the leading noncommunicable health problem worldwide.^[5]^

Pakistan has been ranked 7th in diabetes disease burden in the world, it is projected to reach 15% by 2030, and if the present scenario continues, Pakistan is expected to move to top 4th place.^[6–8]^ According to the current statistics of International Diabetes Federation (IDF), in Pakistan there are 7.028 million cases of adults (20–79 years) with diabetes, with 6.9% prevalence rate and 2.928 million undiagnosed adults with diabetes.^[4]^

T2DM is the most prevalent (>90%) type of diabetes,^[9]^ requiring patients to adopt specific life style modifications in addition to continued pharmacotherapy. Because of the chronic nature of T2DM, diabetes self-care activities have become an integral component of effective diabetes care around the globe.^[10]^ Glycemic control, measured in terms of glycosylated hemoglobin (HbA1c), has been associated with significant reduction in diabetes-related complications and economic burden.^[11]^

T2DM is a self-managed, chronic metabolic disorder, where patients has to take major responsibility to manage their condition by adopting healthy self-care behaviors. The consequences of these self-care behaviors can directly affect health. Managing T2DM requires a huge amount of time, modifications in lifestyle, and confidence to do this. Because of the high cost associated with controlling diabetes, health care providers are now taking an active role in the provision education for diabetes self-management.^[12]^

Educational interventions can play an important role to improve patients’ knowledge and skills regarding diabetes self-management. Systematic reviews of interventions addressing diabetes self-management indicate that the diabetes education courses enhance knowledge, self-care behaviors, and reduction in HbA1C.^[13–16]^ Structured self-management education programmes can be delivered on a one-to-one basis or to groups of people with diabetes and should be facilitated by trained educators. The aim of the education is not only to improve knowledge and skills, but also to motivate a person to make and sustain lifestyle changes, by giving them the confidence to make their own treatment/lifestyle choices on a day-to-day basis.

Educational intervention on self-management can be delivered as group education or individually as one-to-one approach. Sturt et al. reported that an individualized approach with the help of a diabetes guide and telephonic support has demonstrated clinically significant reductions in A1c levels, for T2DM patients with poor glycemic control.^[17]^ Health literacy has been reported as a significant factor influencing the patients’ glycemic control.^[18]^ Clinical outcome can be achieved by addressing patients’ literacy. Pharmacist-based educational interventions are associated with improved medication adherence and glycemic control.^[19–21]^ A review of 16 pharmacist-based interventions resulted in an absolute reduction of HbA1c with an average of 0.62%.^[20]^ Similarly, in an another review, a reduction in A1c levels up to 2.1% was demonstrated by 20 pharmacist-based interventions.^[13]^ A study examining the effectiveness of a free rural clinic for diabetes patients managed by pharmacists found that patients who received education on diabetes, lifestyle modifications, and management of diabetes drug therapy had significant reductions in A1c (≥ 1%) after 24 months.^[22]^

Keeping in view the importance educational interventions in diabetes self-care and clinical outcomes, we designed a face-to-face short-term (6 months) pharmacist-led educational intervention model in poorly controlled T2DM patient (HbA1c >7%) in Pakistan. In this study, we will investigate the mean change in HbA1C at 6 months after the baseline, and a comparison will be made with a similar comparator group of T2DM patients receiving standard diabetes care. In addition, comparison between self-care activities and disease knowledge between the groups will also be studied.

## 2 Methods

### 2.1 Study design

**Figure 1:**
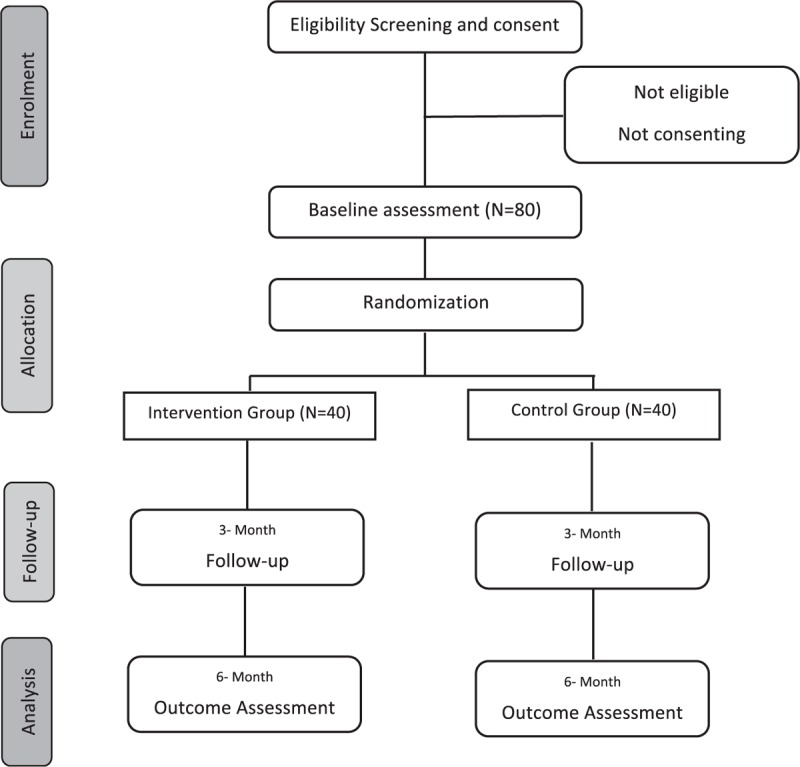
Study Flow Diagram.

This is a 24-week open label, parallel group, prospective randomized controlled trial. In this study, the impact of a pharmacist-based, face-to-face educational intervention will be investigated in adult Pakistani people with poorly controlled (HbA1c >7%) type 2 diabetes. The study flow is presented in (Fig. 1).

### 2.2 Study settings

This 6-month randomized controlled study will be conducted in a tertiary care diabetes care clinic of Capital Hospital (CDA), located in Islamabad, Pakistan. The recruitment and data collection will be performed between October 2017 and March 2018. Setting for pharmacist educational intervention in this study will be a private counselling room at CDA diabetes outpatient clinic.

### 2.3 Study population

Eligibility criteria for the study includes poorly controlled (HbA1c>7%) T2DM, adult patients (age ≥ 30 years) regardless of gender; speak and understand Urdu and / or English languages; having no significant comorbidity; being not involved in any trial/study related to diabetes during last 3 months and able to attend regular visits. Participants will be excluded if they are of other types of diabetes (gestational diabetes, T1DM); unable to answer the questionnaire independently or having hearing, vision, or cognitive impairments.

### 2.4 Sample recruitment procedures

Patients will be identified from the hospital record and who will meet the inclusion criteria of the study will be invited telephonically for participation in the study. If the patient is willing to participate in the study, an appointment will be set in the outpatient counselling room of the pharmacist at CDA hospital and purpose of the study will be explained to the patient and his/her written informed consent will be obtained and baseline assessments will be done. Simple random sampling technique will be used from a list of random numbers of eligible patients which will be compiled by using the patients’ hospital identification numbers. After recruitment, the patients will be requested to handpick an envelope from the basket indicating allocation to either control or intervention group with 1:1 randomization. AB will generate the random allocation sequences, MSN will enrol participants and HS will assign participants to intervention and control groups.

### 2.5 Study aims

#### 2.5.1 Primary aim

To investigate the effectiveness of a pharmacist-delivered diabetes management educational program on glycemic control (HbA1c) after completion of intervention (24th week).

#### 2.5.2 Secondary aim

To examine the impact of a pharmacist-delivered diabetes management educational program on self-care behaviors and patients’ diabetes knowledge, by using Urdu versions of 24-item Diabetes knowledge Questionnaire (DKQ) and 16-item Diabetes Self-management Questionnaire (DSMQ).

#### 2.5.3 Sample size calculations

Study sample size is calculated based on previous studies ^[23–28]^ for detecting the difference of 1% reduction for HbA1C (effect size) with standard deviation of 1.4%, at 6 months in the intervention group. A significance level of 0.05 is considered with study power of 80%. Based on these numbers, the calculated sample size is 62 (31 for experimental group and 31 for controlled group); however, a sample of 80 patients is assumed to be sufficient to compensate for a 25% attrition rate.

#### 2.5.4 Study procedure

The participants will not be informed of their groups. After the initial assessment at baseline, the control group will receive usual care, whereas the interventional group will be provided with the structured intervention. A qualified pharmacist (researcher) will educate the patients in the interventional group about disease (diabetes), its symptoms, clinical goals, self-care activities (self-monitoring of blood glucose, physical activity, importance of regular medication in-take and healthy diet), and reducing risks, whereas the control group patients (N = 40) will receive the usual care.

The pharmacist will provide face-to-face diabetes management education (approximately 30 minutes duration) to the patients in the intervention group at their first visit, follow-up visit (12th week), and 4 telephone (4th, 8th, 16th, and 20th week) and will recommend physician visits when necessary. In addition to face-to-face educational intervention patients in the intervention group will also be provided informatory brochures about self-care activities, and a log book to keep record of their self-monitored blood glucose levels. The educational intervention and patient informatory material has been designed according to American diabetes Association (ADA) guidelines, the details of are presented in Table 1.

**Table 1:**
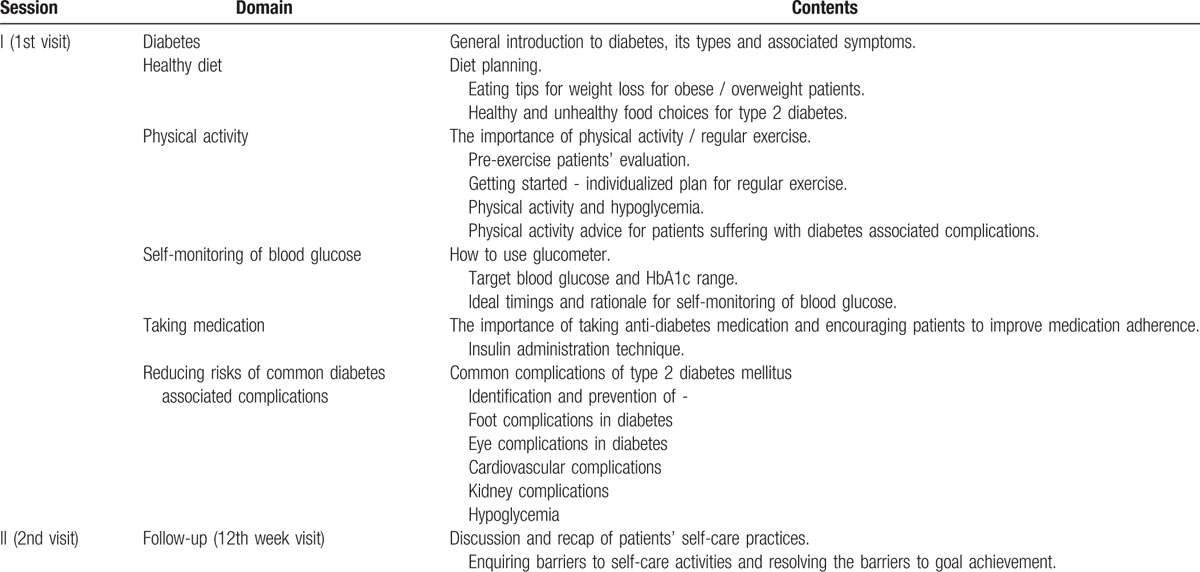
Module for educational intervention.

Details about patients’ (intervention and control group) demographics and lab profile (HbA1c, random blood sugar, fastening blood sugar, etc.) will be obtained by the pharmacist at first visit. At baseline and end of the trail (24th week), the diabetes disease knowledge and self-care activities of the patients in both groups (intervention and control) will be assessed by using 2 prevalidated questionnaires in Urdu language [24-item Urdu version of DKQ and 16-item Urdu version of DSMQ]. Pharmacists can assist the patient in completing questionnaires, but will not provide any help in answering the questions, as the same questionnaires will be used repeatedly. The details of study schedule for data collection are presented in Table 2.

**Table 2:**
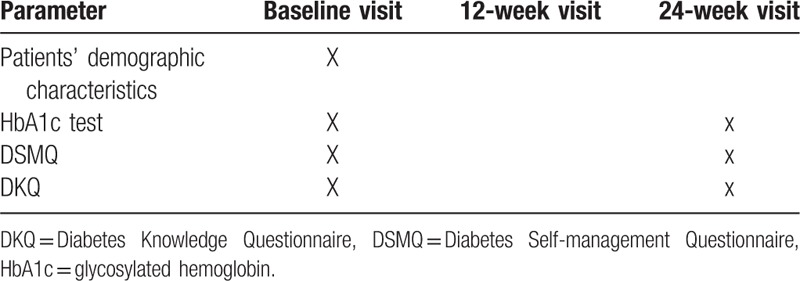
Study schedule for data collection for intervention and control groups.

### 2.6 Measurement tools

#### 2.6.1 Diabetes knowledge score

DKQ, developed for the Starr County Diabetes Education Study, has been translated and validated in Urdu language in Pakistan.^[29]^ DKQ is a 24-item questionnaire. The DKQ scoring involves summing up the scores of all the correct items of each participant, where higher score will indicate better patient's diabetes knowledge. One point will be given to each correct answer and no point for the incorrect option.

#### 2.6.2 Self-care practices

DSMQ will be used to measure the patient's self-care activities. DMSQ was originally developed to evaluate diabetes self-care behaviors associated with glycemic control within common therapeutic options for adult patients of type 1 and type 2 DM.^[30]^ Respondents will be asked to rate their specific self-care activities of last 3 months on a 16-item DSMQ. This questionnaire involves a four point likert scale (ranging from 0 – ‘does not apply to me’ to 3 – ‘applies to me very much’). There are 4 subscales of this study tool; namely, ‘Glucose Management’ (GM), which is comprised of 5 statements: 1, 4, 6, 10, 12, ‘Dietary Control’ (DC): comprised of 4 statements: 2, 5, 9, 13, ‘Physical Activity’ (PA): comprised of 3 statements: 8, 11, 15, and, ‘Health-Care Use’ (HU): comprised of 3 statements: 3, 7, 14. The last item (item 16) asks the respondents to rate their overall diabetes self-care, hence its score is included only in the ‘Sum Scale’ (summation of all of the 16 items).

The scoring process of the DSMQ involves, adding up the scores of all 16 items after reversing the scores of 9 negatively keyed statements. Higher scores will represent more effective self-care. Finally DSMQ scores will be transformed to a scale ranging from 0 to 10, where a score of 10 will indicate the most effective self-care behavior.

#### 2.6.3 Statistical methods

The analyses will be performed using SPSS 22.0.0 (SPSS Inc., Chicago, IL, USA). Frequencies and descriptive statistics will be used for patients’ demographic presentation, while means and standard deviations will be calculated for the continuous variables and group differences will be analyzed by using Pearson chi-square test for categorical variables. Kolmogorov-Smirnov test will be applied to check the distribution of data. Independent *t* test will be used in case of normally distributed data, whereas, Mann–Whitney *U* test for non-normal distributions. Primary outcomes of the educational intervention, which are changes in HbA1C levels, self-care activities scores (DSMQ) and disease knowledge scores (DKQ) will be analyzed by using one-way analysis of variance (ANOVA) for normally distributed data and Kruskal Wallis Test for non-normally distributed data. *P* < 0.05 will be considered as significant for all analysis.

#### 2.6.4 Ethics and dissemination

This study protocol has been approved by Human Research and Ethics committee of Capital Hospital, Islamabad, Pakistan, and, Australian New Zealand Clinical Trials Registry (Registration No. ACTRN12617001327370). Informed written consent will be obtained from all the study participants, after explaining them the purpose and procedure of the study. Participants can withdraw from the study willingly at any point of the study. Confidentiality of patients’ response and data will be assured. All procedures performed in this study involving human participants are in accordance with the ethical standards of the institutional and/or national research committee and with the 1964 Helsinki declaration and its later amendments or comparable ethical standards. After completion of the trial a manuscript with detailed results will be published in a peer review journal.

## 3 Discussion

Previous trials of pharmacist-based educational interventions targeting self-management have demonstrated reductions in HbA1c levels in T2DM in various countries.^[31–34]^ This article describes the study protocol of a RCT evaluating the effectiveness of a short educational intervention to improve self-care activities, disease knowledge and glycemic control in poorly controlled T2DM patients in Pakistan. In 2010, Qayyum et al. observed a clinically significant effect of diabetes self-management education (DSME) on HbA1c reduction in type 1 diabetes children in Pakistan.^[35]^ To date, pharmacist-led educational intervention has not been formally tested in T2DM patients in Pakistan. Therefore, this study will provide valuable information on the effectiveness of this intervention in terms of HbA1c reduction, improvements in disease knowledge and self-care activities.

This study protocol addresses the need to investigate the impact of new educational approaches to deliver a regular diabetes self-management support program. The research will also demonstrate the practicality of such educational intervention on a larger scale. We hypothesized that intervention arm patients will exhibit improvements in HbA1c, self-care activities and disease knowledge in comparison to the control group participants. Thus, we can assume that if 6 months intervention can produce significant reduction in HbA1c, it will also result in beneficial effect on patients’ long-term outcomes.

A systematic review by Wubben et al. (2008) on impact of pharmacist 18 interventional studies on diabetes patients demonstrated a significant reduction in HbA1c, ranging from +0.2 to -2.1% in intervention group as compared to control group.^[13]^ A similar effect has also been reported in a recently published systematic review by Pousinho et al. (2016), where 24 studies out of 26 pharmacist-based interventions in the management of T2DM showed a greater reduction in HbA1c (difference ranging from -0.18% to -2.1%) in interventional group.^[14]^ Improvement in self-care activities has been reported in several pharmacist-based RCT studies.^[24,33,36]^

To date, limited studies have been conducted to address the association of self-care activities with glycemic control in T2DM patients in Pakistan. From a generalizability perspective, this study will recruit a diverse population across urban and rural Islamabad and Rawalpindi, and can be generalizable to other areas in Pakistan. If the study hypotheses are confirmed, the educational module and material could be applied in diabetes health care, resulting in reduction of diabetes related complications.

### 3.1 Strengths and limitations

This interventional study is first of its kind to be conducted in Pakistan involving randomized control design. This study outcome will help to demonstrate the value of implementing a pharmacist-led educational intervention at diabetes care settings to improve self-care practices and clinical outcomes among Pakistani T2DM patients. A possible limitation of the study is the short duration (6 months intervention), which may not make it possible to examine intervention influence on diabetes related complications. Additionally, a 6-months intervention may result in high drop-out rate. To reduce the chances of drop-out, the researcher will facilitate patients in making appointments for follow-up assessment, and an additional 25% sample population will be recruited to compensate the drop-out.

## Acknowledgments

The authors thank the colleagues from the CDA hospital for their efforts in designing of this project. In addition, we would like to thank Global Asia 21 platform.

## 4 Author contributions

**Conceptualization:** A. Bukhsh, T.M. Khan.

**Data curation:** A. Bukhsh, H. Sajjad, M.S. Nawaz.

**Formal analysis:** A. Bukhsh, T.M. Khan.

**Investigation:** A. Bukhsh, H. Sajjad, M.S. Nawaz.

**Methodology:** A. Bukhsh, T.M. Khan.

**Project administration:** A. Bukhsh, T.M. Khan.

**Supervision:** A. Bukhsh, T.M. Khan.

**Validation:** A. Bukhsh, H. Sajjad, T.M. Khan.

**Visualization:** A. Bukhsh.

**Writing – original draft:** A. Bukhsh.

**Writing – review & editing:** T.M. Khan.
